# Editorial: Extra-Corporeal Membrane Oxygenation in Pediatric Cardiac Patients

**DOI:** 10.3389/fped.2018.00204

**Published:** 2018-07-30

**Authors:** Antonio F. Corno, Tara Karamlou

**Affiliations:** ^1^Cardiovascular Research Center, University of Leicester, Leicester, United Kingdom; ^2^East Midlands Congenital Heart Center, University Hospitals Leicester, Leicester, United Kingdom; ^3^Mayo Clinic and Phoenix Children's Hospital, Phoenix, AZ, United States; ^4^Phoenix Children's Hospital, Phoenix, AZ, United States

**Keywords:** thrombosis, multiorgan faliure, ECMO, ventricular assist device, life support

Bartlett pioneered the use of Extra-Corporeal Membrane Oxygenation (ECMO) for prolonged cardio-respiratory support in critical patients in the 1970's (1), reporting on the first pediatric patient to survive: a young child with myocardial dysfunction after surgery for transposition of the great arteries (2). Early clinical successes led to increased use of ECMO to support children with respiratory and cardiac failure. ECMO was initially used for resuscitation after cardiac arrest in pediatric patients, and then as peri-operative stabilization for palliative and corrective procedures for congenital heart defects. Despite the recent advances in Ventricular Assist Device technology, ECMO remains the most commonly used system of mechanical circulatory and respiratory assistance in pediatric cardiac patients. The advantages of ECMO include: its familiarity among the caregivers involved with the management of pediatric patients with complex congenital heart defects, the capability of providing cardiac (bi-ventricular) and respiratory support, the availability across all pediatric age and body weight groups and the relatively low costs. Furthermore, ECMO is ideal suited for pediatric patients with a combination of cardiac and respiratory failure, frequently occurring after repair of complex congenital heart defects, and in cases of rapid deployment during a cardio-circulatory arrest. Disadvantages of ECMO include the need of a dedicated team of specialists, intensive care monitoring and the risk of major potential complications such as bleeding, thrombosis, infections and multi-organ failure. In this Research Topic several experts in the field report on the state of the art knowledge on the topic.

Burke and McMullan contribute a very extensive and precise review of their experience with Extra-Corporeal Life Support (ECLS) for pediatric heart failure, including the utilization as peri-operative support as bridge to heart transplant. Very appropriately, they present the extracorporeal cardio-pulmonary resuscitation as one of the most significant advancements in the field of ECLS (Burke and McMullan). In addition to a clear list of contraindications, a very detailed list of the potential complications was given, including hemorragic episodes, central nervous system injuries, pericardial tamponade, pneumothorax, metabolic acidosis, seizures, infections, renal failure, failure of the oxygenator, and limb ischemia (Burke and McMullan).

Di Nardo et al. contribute review of ECLS, with particular attention to current indications, pre-operative stabilization, peri-operative support and use beyond the peri-operative period (Di Nardo et al.).

The contraindications, as well as the details to facilitate the cannulation in neonate and small patients are reviewed (Di Nardo et al.). An important contribution was given with a list of special indications for ECLS: intractable arrhythmias, extracorporeal cardio-pulmonary resuscitation, and use in the presence of “functionally” uni-ventricular type of circulation (Di Nardo et al.).

Their very strong message was that every attempt should be done to start ECLS “urgently” rather than “emergently,” before the presence of dysfunction of end organs or circulatory collapse (Di Nardo et al.).

Harvey contributed a useful and practical review on cannulation, based on his very extensive clinical experience. Pure cannulation considerations about the design of the available cannulas and the type of required support are discussed (Harvey). For the Veno-Arterial (V-A) support suggestions were given for difficult cannulation, with special considerations particularly for neonates, and for the central cannulation for V-A support. For the Veno-Venous (V-V) support a list of potential complications was presented, with suggestion about their prevention and treatment (Harvey). A separate part was dedicated to the surgical cannulation of the femoral vessels for larger size patients, with attention to the distal limb perfusion, as well as to the vessel sparing cannulation (Harvey). Finally, all aspects related to the need of left heart venting were presented, for both situations of open and closed chest left heart venting (Harvey).

Broman and Frenckner contribute an interesting article on the transportation of critical patients on ECMO. After an accurate report of the background, they gave a carefully detailed description of the logistics of the transport, including the requirements for the transport vehicle, the equipment and the personnel (Broman and Frenckner). Special situations were also taken in consideration, such as international transportations, environmental and climate issues, and working environment (Broman and Frenckner).

Ryerson and Lequier contribute a review of one of the most important and difficult issues of the ECLS: the management and monitoring of the anticoagulation. They very appropriately started by specifying than anticoagulation *per se* is more complex in neonates and infants, and anticoagulation on ECLS provides an additional layer of complexity (Ryerson and Lequier). Furthermore, heparin is only an indirect anticoagulant, and the standard monitoring is hugely imperfect. Then they analyzed the role of activated clotting time, activated partial thromboplastin time, anti-Xa assay, viscoelastic testing, and antithrombin replacement (Ryerson and Lequier). Alternative options of anticoagulation were considered, such as direct thrombin inhibitors, new oral anticoagulants, and factor XIIa inhibitors. They provide an useful review on the prevention and management of bleeding and thrombotic complications during ECLS: definition and criteria of significant bleeding, role of antifibrinolytic therapy, activated factor VII, thrombotic complications in the patient and in the circuit, heparin-induced thrombocytopenia, and finally the optimal blood products replacement (Ryerson and Lequier).

In the last article of this Research Topic Nair and Oishi report their experience with the management of patients with single-ventricle pathophysiology and acute respiratory distress syndrome (ARDS), with V-V ECLS (Nair and Oishi). After an extensive introduction and explanation of the role of the ECLS in the presence of respiratory issues, they focused on ECLS in patients with “functionally” uni-ventricular hearts, discussing the role of V-V vs. V-A support. Special considerations were proposed for patients with “functionally” uni-ventricular hearts requiring V-V support because of ARDS: in particular (a) timing of cannulation, (b) type and positioning of the cannula, (c) bleeding, thrombosis and anticoagulation, (d) ECLS flow in these patients, (e) lung rest settings, and finally (f) weaning from ECLS.

We strongly believe that this collection of articles on ECMO will be very useful to all people involved in the care of pediatric patients requiring cardiac and/or respiratory support.

## Author contributions

AFC and TK edited this Research Topic and this editorial was written by AFC.

### Conflict of interest statement

The authors declare that the research was conducted in the absence of any commercial or financial relationships that could be construed as a potential conflict of interest.
